# Significance of Fibrillin-1, Filamin A, MMP2 and SOX9 in Mitral Valve Pathology

**DOI:** 10.3390/ijms25179410

**Published:** 2024-08-29

**Authors:** Carmen Elena Opris, Horatiu Suciu, Ioan Jung, Sanziana Flamand, Marius Mihai Harpa, Cosmin Ioan Opris, Cristian Popa, Zsolt Kovacs, Simona Gurzu

**Affiliations:** 1 Department of Adult and Children Cardiovascular Recovery, Emergency Institute for Cardio-Vascular Diseases and Transplantation, 540139 Targu Mures, Romania; carmenchincisan@gmail.com; 2 Department of Pathology, George Emil Palade University of Medicine, Pharmacy, Science and Technology, 540139 Targu Mures, Romania; janos.jung@umfst.ro; 3 Department of Surgery, George Emil Palade University of Medicine, Pharmacy, Science and Technology, 540139 Targu Mures, Romania; horatiu.suciu@umfst.ro (H.S.); flamind.sanzi@gmail.com (S.F.); marius.harpa@umfst.ro (M.M.H.); nymcos1978@gmail.com (C.I.O.); 4 Romanian Academy of Medical Sciences, 030173 Bucharest, Romania; 5 Faculty of European Studies, Babes-Bolyai University, 400006 Cluj-Napoca, Romania; statasales@token.com.ro; 6 Department of Biochemistry and Environmental Chemistry, George Emil Palade University of Medicine, Pharmacy, Sciences and Technology, 540139 Targu Mures, Romania; zsolt.kovacs@umfst.ro; 7 Research Center for Oncopathology and Translational Medicine (CCOMT), George Emil Palade University of Medicine, Pharmacy, Science and Technology, 540139 Targu Mures, Romania

**Keywords:** Fibrillin 1 (FBN1), Filamin A (FLNA), matrix metalloproteinase 2 (MMP2), SRY-box transcription factor 9 (SOX9), mitral valve

## Abstract

Genetic factors play a significant role in the pathogenesis of mitral valve diseases, including mitral valve prolapse (MVP) and mitral valve regurgitation. Genes like Fibrillin-1 (FBN1), Filamin A (FLNA), matrix metalloproteinase 2 (MMP2), and SRY-box transcription factor 9 (SOX9) are known to influence mitral valve pathology but knowledge of the exact mechanism is far from clear. Data regarding serum parameters, transesophageal echocardiography, and genetic and histopathologic parameters were investigated in 54 patients who underwent cardiovascular surgery for mitral valve regurgitation. The possible association between Fibrillin-1, Filamin A, MMP2, and SOX9 gene expressions was checked in relationship with the parameters of systemic inflammatory response. The mRNA expression levels (RQ—relative quantification) were categorized into three distinct groups: low (RQ < 1), medium/normal (RQ = 1–2), and high (RQ > 2). Severe fibrosis of the mitral valve was reflected by high expression of FBN1 and low expression of MMP2 (*p* < 0.05). The myxoid degeneration level was associated with the mRNA expression level for FBN1 and a low lymphocyte-monocyte ratio was associated with an increased mRNA expression of FBN1 (*p* < 0.05). A high number of monocytes was associated with high values of FBN1 whereas the increase in the number of lymphocytes was associated with high levels of MMP2. In addition, we observed that the risk of severe hyalinization was enhanced by a low mRNA expression of FLNA and/or SOX9. In conclusion, a lower FLNA mRNA expression can reflect the aging process that is highlighted in mitral valve pathology as a higher risk for hyalinization, especially in males, that might be prevented by upregulation of the *SOX9* gene. FBN1 and MMP2 influence the inflammation-related fibrotic degeneration of the mitral valve. Understanding the genetic base of mitral valve pathology can provide insights into disease mechanisms, risk stratification, and potential therapeutic targets.

## 1. Introduction

Mitral valve disease is known as one of the most common forms of cardiac disease, affecting 1–2% of the population [1]. Understanding the pathomechanism and the factors involved in aggravating mitral insufficiency is extremely important in the treatment and prevention of valvular pathology. Although it is known that genetics might play a role in the pathology of mitral valve prolapse, whose incidence occurs in 2–3% of the population [2], limited data exists about the genetic background of mitral valvulopathy.

To understand mitral valvulopathy, it is important to look closer at the gene-related embryology that was recently synthesized by our team in a review-type article [3]. The most common genes proved to influence cardiac development are the transcription factors NKX2-5, T-box transcription factor 5 (TBX5), and GATA-4. On the other hand, the SRY-box transcription factor 9 (SOX9) and Filamin A (FLNA) proved to play roles in the remodeling of the mesenchymal tissue of the cardiac cushions, and cardiovascular remodeling, during embryology. In adults, they also influence valvular aging, but the exact mechanism is not known yet. As matrix-metalloproteinases (MMP) such as MMP-2 are also known to be pro-apoptotic and modulate the epithelial and mesenchymal tissue plasticity, especially in tumors [4], we hypothesized for the first time in the literature, that the valvular degeneration might be influenced by the possible interaction of the three genes. To have a complete picture of the examined micromedium and extracellular matrix (ECM), we added the extracellular glycoprotein Fibrillin-1 (FBN1) to the profile.

## 2. Results

### 2.1. Clinicopathological Parameters and mRNA Expression from Blood Samples

There were 54 patients included in this observational study. Most patients were men (*n* = 31; 57.41%), from urban environments (51.85%), and the age range of the entire cohort was 20–76 years old. The men’s mean age was 61.3 and the women’s mean age was 62.4 years.

The tissue FBN1 mRNA expression was low in 12 of the 54 patients (22.2%), medium/normal expression in 21 (38.9%), and high in the remaining 21 patients (38.9%). The FLNA expression was low in 14 patients (25.9%), medium in 23 patients (42.60%), and high in 17 patients (31.5%). The MMP2 expression was low in 37 patients (68.5%), medium in 16 (29.6%), and only one patient showed high MMP2 (1.9%). The SOX9 expression was low in two patients (3.7%), medium in 17 (31.5%), and high in 35 patients (64.8%).

Firstly, we analyzed the association between the sex of the patients and mRNA expression (Table 1), and then the sex and age relation with the mRNA expression using multinomial logistic regression: relative risk ratio (RRR) (Table 2).

SOX9 gene expression was not influenced by the patient’s age or sex. FBN1 tended to be lower in females, whereas MMP2 was more downregulated in males compared with females (RRR 0.14, *p* = 0.005). None of these mRNA expressions were influenced by the age of the patient (Table 1 and Table 2).

The chances of having high FLNA expression, compared to medium expression, were lower for men compared with women (RRR 0.18), especially for elderly patients (RRR 0.91) (Table 1 and Table 2).

The correlation between the mRNA expression level and the environment of residence was also checked but it did not indicate any statistical significance.

The echocardiographic parameters were evaluated by transthoracic echocardiography. As most patients had normal left ventricle ejection fraction (≥50%), both preoperative (*n* = 48; 88.89%) and postoperatively (*n* = 42; 77.78%), the interaction by categories in conjunction with genetics was statistically insignificant (we used the multinomial logistic regression test). In mitral insufficiency, the ejection fraction can be supernormal due to the increased preload from volume overload [5]. We analyzed whether there is a difference in ejection fraction (EF) associated with mRNA expression in both tissue and blood samples. The ejection fraction increased by 8.84 units (%) in the presence of high MMP2 expression in blood samples compared to normal expression (Coef 8.8, *p* = 0.012) (Table 3). Regarding the tissue samples, the analysis was inconclusive.

We further investigated the influence of mRNA expression in valve tissue and blood samples on the left ventricle end-diastolic diameter, using it as a marker of ventricular remodeling (Table 4).

1. The end-diastolic LV diameter decreases with 9.29 units (mm) in the case of low FBN1 tissue expression, compared to the normo-expression (coef −9.29, *p* < 0.05)

2. The end-diastolic LV diameter increases with 4.56 units in the case of low FLNA tissue expression, compared to normo-expression (coef 4.56, *p* < 0.1)

3. The end-diastolic LV diameter increases with 5.7 units for the low expression of tissue MMP compared to normo-expression (coef 5.7, *p* < 0.05).

Regarding the mRNA expression in blood sample, we found no correlation between the gene expression and the remodeling of the left ventricle.

### 2.2. Inflammatory Markers and mRNA Expression from Blood Samples

Testing the relationship between mRNA expressions and inflammatory markers using linear regression, we assumed that as the genes (and their variations in expression levels) are the basis of physiological traits, influencing the synthesis or regulation of various biomolecules (lymphocytes, monocytes, etc.), their role might be reflected in the analyzed biomarkers. Therefore, we considered the genetic expression as the independent variable and the biomarker as the dependent (determined) variable. We considered normal mRNA expression as a reference level (Table 5).

Interpretation of the statistical data (Table 5) showed that FLNA and SOX9 did not influence any of the inflammatory-related parameters. A lower lymphocyte-to-monocyte ratio (LMR) reflected an increased expression of FBN1 (*p* = 0.031), whereas increase in the number of monocytes was associated with high values of FBN1 (*p* = 0.04). The increase in the number of lymphocytes was also associated with high levels of MMP2 expression (*p* = 0.068, significance at *p* < 0.1 level).

### 2.3. Histopathological Features and mRNA Expression from Blood Samples

The examined histopathological parameters included fibrosis, hyalinization, and myxoid degeneration. They were classified as low and high degree. In the adjustment, we also considered the age factor for a proper statistical interpretation (Table 6).

We first analyzed the adjusted values and concluded that a low expression of MMP2 reflected a higher chance of severe fibrosis (aOR = 10.462, *p* = 0.029). A low expression of FLNA, compared with normal expression, was associated with a higher risk for severe hyalinization (aOR = 9.883, *p* = 0.062—the significance is weaker, at a confidence level of 90%—*p* < 0.1). An increased expression of SOX9 reflected a lower chance for severe hyalinization (aOR = 0.182, *p* = 0.085 at *p* < 0.1 level). Age, analyzed in conjunction with mRNA expression, is an indicator of increased chances of severe hyalinization (aOR = 1.105, *p* = 0.052) (Table 6).

If the aforementioned data are taken together, as FLNA expression tended to decrease in males and in parallel with age (Table 1 and Table 2), and the hyalinization was proved to be more severe in conjunction with age (Table 6), it can be concluded that a lower FLNA mRNA expression can reflect the aging process that is seen, in mitral valve pathology, as an increased risk of hyalinization, especially in males.

If we analyze the “raw” values, we see that the high value of FBN1 compared to the average (normal) value is presented as a statistically significant indicator for severe fibrosis (*p* = 0.051, OR = 9.23). This observation is still at the limit of the conventional level, *p* = 0.05 (borderline). Also, the low value of MMP2 expression is a strong predictor for severe fibrosis (OR = 7.3, *p* = 0.013); MMP2 proves to be a significant predictor in conjunction with the other studied values (adj OR = 10.462, *p* = 0.029).

The low level of FBN1 compared to the normoexpression of the gene appears as a significant indicator of the low severity of myxoid degeneration (OR = 0.196—the chance is low, *p* = 0.047). Conversely, we can say that the normoexpression is significantly associated with the increased severity of myxoid degeneration.

### 2.4. mRNA Expressions from Blood Versus Tissue Samples

To have a full picture of the genetic background, we analyzed the possible association between mRNA expression detected from blood and that determined from the frozen mitral valve tissue. We applied the Fisher exact test of independence, the method most suitable for the type of data we have and their distribution, on the pairs of blood samples vs tissue samples. The null hypothesis (H0) of independence test was used (Table 7).

The null hypothesis was rejected in all cases indicating—what we expected—that the values are associated.

### 2.5. Gene-Gene Interactions in Blood and Tissue Samples

We also considered correlations between the expressions of the four examined genes (Table 8).

**Figure 1:**
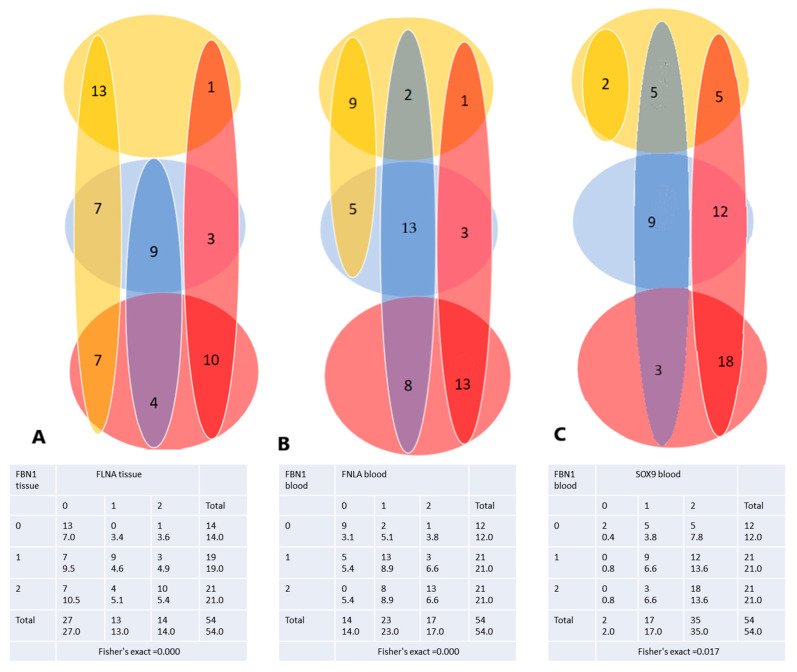
Venn diagram: (**A**). FBN1 vs. FNLA—from tissue samples, (**B**). FBN1 blood vs. FNLA blood, (**C**). FBN1 blood vs. SOX9 blood. Color code: yellow: mRNA expression 0, blue: mRNA expression 1, red: mRNA expression 2. Fisher’s exact test: Key: frequency/expected frequency.

The association between variables FBN1 and FMNA was assessed using Fisher’s exact test, which yielded a *p*-value of less than 0.001 (*p* < 0.001). This result indicates a statistically significant association between the two variables. Unlike the comparison of the results on the tissue sample, it appears in the blood sample that FBN1 and SOX9 are associated, so they have a causal relationship. The results are expressed by way of a Venn diagram (Figure 1).

The Venn diagram demonstrates that a low FLNA profile is associated with a low FBN1 profile, and a high FBN1 profile is associated with a high FLNA profile in the tissue sample. We see, for example, that the method “predicts” the value 7 at the low-low intersection (for a distribution of pairs that ensures independence), but in fact, we have 13 cases of overlap. If we run the multiple logistic regression on the two variables (FLNA depending on FBN1) we get a result indicating that the high expression of FBN1 compared to the normal level is an indicator of the high expression of FLNA compared to the normal expression (RRR = 7.5, *p* = 0.024).

In the case of FBN1 and FLNA collected from blood, the situation looks about the same. As can be seen, the actual values exceed the “predicted” values at low-low and high-high expression interactions. Logistic regression indicates in this case an increased RRR for low FLNA compared to normal in the case of low FBN1 expression (RRR = 11.7, *p* < 0.009) and an increased RRR for high FLNA expression in the case of high FBN1, compared to the normal expression (RRR = 7, *p* = 0.013).

Also, multiple logistic regression indicates increased RRR for SOX9 high expression, when FBN1 is high, compared to the normal expression (RRR = 4.5, *p* = 0.049).

## 3. Discussions

Mitral regurgitation affects about 24.2 million people around the world, being the third most common valvular disease [6]. Its incidence varies depending on several factors such as age, sex, associated cardiovascular conditions, and the presence of an inflammatory environment. The main causes of mitral regurgitation are mitral valve prolapse, rheumatic disease, infective endocarditis, mitral annulus calcification, cardiomyopathies, and ischemic heart disease [7,8,9]. Depending on the etiology, mitral insufficiency can be primary or secondary.

In primary mitral regurgitation, the valvular structures are directly affected. In the last 25 years, the etiology of valvular diseases has changed, moving from rheumatic damage to the predominance of degenerative changes. Thus, myxomatous degeneration predominates in young people, causing prolapse or “flail” of the mitral valves. This is mainly due to genetic predispositions [6] that might be influenced by FBN1 gene, while fibromatous degeneration and hyalinization predominate in the elderly [7] and, as the present study shows, seem to be modulated by FLNA gene.

In Europe and the USA, the most common cause of mitral regurgitation is attributed to secondary or functional mitral regurgitation [8,9,10]. It occurs secondary to other cardiac changes such as myocardial ischemia, with deformation of the geometry of the ventricular cavity at the level of the insertion base of the papillary muscles, dilation of the mitral ring, in dilated cardiomyopathy, or dilation of the left atrium, in chronic atrial fibrillation.

As many recent studies revealed, gene expression differs in blood and tissue samples [11,12,13]. In this study, we explored the possible influence of the genetic background on mitral valve structure and function. Four genes, FBN1, FNLA, MMP2, and SOX9, were examined from tissue and blood samples. Unlike the comparison of the results on the tissue sample, it appears in the blood sample that FBN1 and SOX9 are associated, so they have a causal relationship. We proved that parallel determination, from blood and tissue, might add supplementary data that can be helpful for understanding the gene involvement in the mitral valve regurgitation, at both tissue and circulating levels. This comparison allowed us to determine whether the presence of mitral regurgitation is associated with changes in mRNA levels in patients with mitral insufficiency.

The results reflect two gene-based tendencies of valve degeneration: one being dictated by aging of the valve and FLNA gene; the second one probably being influenced by the inflammatory environment, especially by the number of circulating lymphocytes and monocytes, with the micromedium probably being modulated by the FBN1 and MMP2 genes.

For the first hypothesis, related to aging, FLNA and SOX9 proved to influence the valve aging process that is reflected by the grade of hyalinization [7]. Our data shows that FLNA gene expression decreases in correlation with the patient’s age, especially in males, and also correlates with the grade of hyalinization, independent of the ejection fraction or the inflammatory environment. It might be a physiological downregulation of FLNA in the elderly that cannot be influenced by external factors. Also, the end-diastolic LV diameter, as a marker of ventricular remodeling occurring in severe mitral valve regurgitation, increases by 4.56 units in the case of low FLNA tissue expression, compared to normo-expression (coef 4.56, *p* = 0.88 observed at a significance level of *p* < 0.1). Many studies have investigated its role in cardiac remodeling after myocardial infarction, and the FNLA may be a target of genetic therapy for preventing ventricular remodeling [14]. On the other hand, a high mRNA expression for SOX9 was an indicator of low hyalinization seen under the microscope. As SOX9 did not show an association with any other parameters, it can be hypothesized that the two examined genes do not influence the inflammatory processes but instead are negatively correlated with the aging process. However, if the SOX9 level is therapeutically upregulated, it might act as a protector for valvular aging.

It is known that SOX9 has an essential role in chondrocyte differentiation, and also promotes the expression of cartilaginous matrix proteins in developing heart valve structures [15]. Endocardial cushions serve as rudimentary valves crucial for guiding blood flow in a single direction within the developing heart tube. As they undergo morphological changes, becoming elongated and slender, the ECM remodels, marking the inception of heart valve formation. Notably, the lateral cushions contribute significantly to shaping the leaflets of the mitral and tricuspid valves, essential components of the mature heart’s valve apparatus [16,17]. As the transcription factor SOX9 is highly expressed in the cardiac cushion mesenchymal tissue [3] and has an important role in the development of heart valves, a higher risk for hyalinization can be found in elderly people with low gene expression for SOX9. A reduced SOX9 expression promotes valve calcification [18].

The second mechanism of valvular degeneration might be related to the systemic inflammatory response that is reflected, according to our statistical data, by LMR and by the number of lymphocytes and monocytes, but not by NLR, C reactive protein-lymphocyte ratio (CLR), platelet-lymphocyte ratio (PLR), or platelet-neutrophil ratio (PNR); none of which showed an association with any of the four examined genes. FLNA and SOX9 were not associated with any of the inflammatory parameters.

In this study, a high FBN1 gene expression reflected a low LMR, a high number of circulating monocytes, and also a high score of fibrosis that was quantified under microscope, especially in males. On the other hand, a high MMP2 gene expression was associated with a high number of circulating lymphocytes but a lower score of fibrosis, especially in females. It can be hypothesized that MMP2-related high serum levels of lymphocytes might be a protective factor against fibrotic degeneration of the mitral valve, but the FBN1 gene influences monocyte proliferation, and its upregulation contributes to the risk of fibrosis. A negative correlation between MMP2 and FBN1 seems to exist. Fibrosis of the mitral valve might be influenced by their interaction which also modulates the systemic inflammatory response of the human body.

The literature data partially sustains our understanding of the FBN1-related valvular modulation. Fibrillins constitute a family of large extracellular glycoproteins that multimerize to form microfibrils, an important structure in the ECM [19]. Microfibrils provide tensile strength in nonelastic tissues and scaffolds for the assembly of tropoelastin in elastic tissues, and act as a regulator of growth factor bioavailability and activity in connective tissues. Fibrillin assembly involves complex multi-step mechanisms to result in a periodical head-to-tail alignment in microfibrils. The fibrillinopathies, a diverse group of connective tissue disorders, are caused by pathogenic variants in FBN1 and FBN2 genes. FBN1 encodes a glycoprotein called Fibrillin-1, a major component of microfibrils in the ECM [20]. Impaired assembly plays a role in the molecular pathogenesis of genetic disorders caused by mutations in Fibrillin-1 (Marfan syndrome) and Fibrillin-2 (congenital contractural arachnodactyly) [21,22,23]. Mutations in the FBN1 gene are associated with cardiovascular, ocular, and skeletal abnormalities [20,23]. In the degenerative process of the mitral valve, a high FBN1 gene expression might influence the severity of fibrosis which is also increased by a high number of monocytes and a low LMR value. LMR, NLR, and PNR proved to be altered in patients diagnosed with mitral valve prolapse [24]. The end-diastolic LV diameter decreases by −9.29 units (mm) in the case of low FBN1 tissue expression, compared to normo-expression (coef −9.29, *p* = 0.003, meaning *p* < 0.05).

The MMPs are a major group of enzymes responsible for the degradation of the ECM, cellular receptors, and cytokines. There are 24 known MMPs which are divided into six groups, based on their structure and function [25]. The activity of MMPs is inhibited by endogenous specific inhibitors called tissue inhibitors of metalloproteinases (TIMPs) [26,27]. MMP2 plays a role in tissue remodeling, wound healing, and various physiological processes such as angiogenesis and inflammation [28,29,30], as is also proven in the present study. Originally known to play pivotal roles in tissue morphogenesis and wound healing, they have been shown to participate in the complex remodeling processes in blood vessels and the myocardium [31]. In the pathogenesis of mitral valve prolapse, significant crosstalk—known as endothelial to mesenchymal transition (EndMT)—between valvular interstitial cells and valvular endothelial cells has been observed in various studies [32,33]. Concerning ECM reorganization, a significant increase in hyaluronic acid content with reduced sulphated glycosaminoglycans may be observed and is related to the TGF-β-induced downregulation of genes belonging to the A disintegrin and metalloproteinases that play a central role in proteoglycans degradation [34,35]. This is responsible for the altered mechanical properties of myxomatous mitral valve leaflets, leading to an increased valve extensibility, and a decrease in leaflet stiffness and failure strain [36]. In humans, a higher level of MMP2 expression is reported in myxomatous valves compared to normal valves, and recognition of a mediating role for MMP2 in the pathogenesis of MVP may have practical implications [37,38]. Our data are in line with the those in the literature but what ours adds is the fact that a low level of MMP2 can induce myxomatous changes in young people and protect against fibrosis by increasing the number of circulating lymphocytes. In our study, the end-diastolic LV diameter increases by 5.7 units for the low expression of tissue MMP compared to normo-expression (coef 5.7, *p* = 0.012, meaning *p* < 0.05).

In our study, we performed mRNA expression analysis for SOX9, MMP2, FLNA, and FBN1, from both blood and tissue samples, in patients with mitral valve pathology. The source of mRNA in the blood can be attributed to several factors, including circulating tumor cells, exosomes, and other extracellular vesicles released from damaged or diseased tissues. In the context of mitral valve pathology, the mRNA detected in the blood likely originates from the mitral valve tissue itself, reflecting the ongoing pathological processes. The release of mRNA into the bloodstream may occur due to cellular turnover, tissue remodeling, and the active secretion of vesicles by cells affected by mitral valve disease. This circulation of mRNA in the blood serves as a potential biomarker, offering insights into the gene expression changes that might influence mitral valve structure and function.

Although genes can modulate any process in the human body, their expression is also influenced by several internal and external factors such as diet, temperature, oxygen levels, humidity, light cycles, the presence of mutagens, lifestyle, exposure to toxins, stress, and physical activity [39]. Further studies are necessary to confirm the hypotheses highlighted in the present paper and partially sustained by the literature data.

## 4. Materials and Methods

### 4.1. Selection of Cases

The examined data were obtained from 54 patients who underwent cardiovascular surgery for mitral valve regurgitation at the Emergency Institute for Cardiovascular Diseases and Transplantation of Targu Mures, Romania, from 2019–2022. Prior to surgery, all patients underwent transesophageal echocardiography. Other data, extracted from the medical records of hospitalized patients, covered demographic information such as age, sex, and associated comorbidities like systemic hypertension, diabetes, stroke, and pulmonary disease. Exclusion criteria encompassed patients over 80 years old, those with chronic inflammatory diseases (e.g., autoimmune disorders), active infections (including acute endocarditis), and malignant tumors. Approval for the study was obtained from the Ethics Committee of the Clinical Emergency Hospital of Targu Mures, Romania (approval no. 2380/9 June 2023). Histopathological examination of the mitral valve was post-operatively conducted on the removed valves for total or partial surgical resection.

### 4.2. Laboratory Data

Biochemical measurements were done using blood that was obtained preoperatively, on the day of admission, following a minimum 8-h fasting period, with cardiac surgery scheduled for the subsequent day. Red blood cell count, white blood cell (WBC) count, and serum levels of lymphocytes, monocytes, neutrophils, platelets, and C-reactive protein (CRP) were determined. WBC levels were analyzed using an automated hematology analyzer employing fluorescence flow cytometry (SYSMEX XS-800; Sysmex Corporation, Kobe, Japan), while CRP levels were assessed utilizing a clinical chemistry analyzer with fluorescence technology (ARCHITECT c4000; Abbott, North Chicago, IL, USA). From these data, the LMR, PLR, PNR, and CLR were calculated. After cardiac surgery, the removed mitral valves were immediately sent to the Pathology Department for subsequent examination.

### 4.3. Echocardiography

Preoperative transesophageal echocardiography was performed on patients in the operation room. Echocardiographic parameters included evaluation of leaflet morphology and motion, sub-valvular structures, annulus size and characteristics, dimensions and function of the left ventricle and left atrium, left ventricular ejection fraction, and the severity of mitral regurgitation. The valve appearance was evaluated as fibrous, cleft, myxomatous, prolapse, or flail. The left ventricle ejection fraction (EF) was determined using transthoracic ultrasound, with EF value divided into normal (≥50 [HFpEF]), slightly reduced (41–49 [HFmrEF]), and reduced (≤40 [HFrEF]) [40].

### 4.4. Microscopic Assessment

In all cases, the removed valves were sent to the Pathology Department as fresh specimens. From each valve, a two-centimeter-sized tissue was preserved at −70 °C for further molecular examinations. The remaining valve was formalin-fixed and paraffin-embedded, and slides were sent for microscopic analysis. Hematoxylin-Eosin (HE) stain was used for overall morphology assessment and Masson’s trichrome was used for evaluation of fibrosis and degree of hyalinization, based on the expansion of the fibrosa and collapse of the spongiosa layer. Cases were graded by two experienced pathologists (SG, IJ) for fibrosis, hyalinization, and myxoid degeneration, achieving a 90% concordance rate. The cases were classified as follows: grade 1 represented mild changes observable at intermediate to high magnification; grade 2 signified moderate changes visible at intermediate magnification; and grade 3 indicated severe changes discernible at low magnification. Cases of grade 1 were categorized as having low degenerative alterations, while those of grade 2 and grade 3 were considered as having high degenerative changes. Any divergent assessments were reanalyzed to reach a final consensus diagnosis.

### 4.5. mRNA Expression Analysis

The significance of mRNA expression of FBN1, FLNA, MMP2, and SOX9, was examined in 54 patients with mitral valve regurgitation who underwent either mitral valve repair or replacement.

*Ribonucleic Acid (RNA) Extraction*: RNeasy Mini Kit (Qiagen, Valencia, CA, USA) was used to isolate RNA from whole blood samples, obtained preoperatively, and from the frozen valvular tissue, that was kept at −80 °C, using the Qiagen’s recommendations for isolation. The RNA extraction process involved homogenization of the sample in a lysis buffer to release RNA, binding RNA to the silica membrane in the spin column, washing to remove contaminants, and elution of pure RNA with RNase-free water provided in the kit.

*Reverse Transcription (cDNA Synthesis)*: Qiagen’s OneStep RT PCR kit was used for reverse transcription, together with SYBR Green ROX PCR Mastermix (Qiagen, Valencia, CA, USA) according to the manufacturers’ recommendations. The reverse transcription process involved: mixing RNA with a reaction buffer containing reverse transcriptase enzymes, nucleotides, and primers; incubation at an appropriate temperature to allow the reverse transcriptase enzyme to synthesize cDNA from the RNA template and inactivation of the reverse transcriptase enzyme to stop the reaction.

*The qPCR process*: This included preparation of a PCR reaction mix containing cDNA, primers specific to the target gene(s), SYBR Green dye (for intercalating dye detection), running the qPCR reaction in a real-time PCR machine (QuanStudio3—Applied Biosystems) which monitors the amplification of DNA during each cycle, and analysis of the amplification curves to determine the threshold cycle (Ct) values which represent the cycle number at which the fluorescent signal crosses a defined threshold. The thermal cycling profile was: denaturation: 95 °C for 20 s to separate the DNA strands, annealing: 65 °C for 20 s for primers to bind to the target DNA, extension: 72 °C for 45 s for DNA polymerase to synthesize new DNA strands with fluorescence measurement at the end of each extension file. In this study, we utilized prefabricated primers from Qiagen for the genes FLNA, FBN1, SOX9, and MMP2. As these primers were commercially obtained, the specific primer sequences are proprietary information of Qiagen, Germany.

*mRNA expression analysis with relative quantification*: A relative quantification method was used to compare mRNA expression levels between different samples. It involved normalizing the expression of the target gene (FBN1, FLNA, MMP2, SOX9—RT qPCR Primer Assay for Human FBN1, FLNA, MMP2 and SOX9 from Qiagen, Germany—used according to manufacturer’s recommendations) to that of a reference gene (GAPDH) and normal samples, comparing the relative expression levels. Relative quantification was used, where the expression levels in patient samples were normalized against the control samples.

*Steps for relative quantification*: These included using appropriate reference genes (housekeeping gene—GAPDH) that are stably expressed across all samples and conditions, performing qPCR for both the target gene and the reference gene in each sample, calculating the ΔCt value for each sample by subtracting the Ct value of the reference gene(s) from the Ct value of the target gene, using the ΔΔCt method to calculate the fold change in gene expression between samples, comparing to a control sample (normal colon tissue). This involved subtracting the ΔCt of the control sample from the ΔCt of each experimental sample. Finally, we calculated the relative expression levels using the formula 2^−ΔΔCt^), which represents the fold change in gene expression relative to the control sample. The mRNA expression level (RQ—relative quantification) was considered as low (RQ < 1), medium/normal expression (RQ = 1–2), and high (RQ > 2), with the study design being similar to our previous research data [41]. To confirm the reliability of the study design, a control group of 10 cases from autopsy was used, from patients under the age of 50 years, who suddenly died without having cardiovascular diseases, chronic inflammatory diseases (e.g., autoimmune disorders), active infections (including acute endocarditis), or malignant tumors. The gene expression analysis, in the control group was done from valvular tissue. In all of the cases the mRNA expression, for all of the genes used in the present study was between 1 and 2.

### 4.6. Statistical Analysis

We performed an extensive statistical analysis utilizing Stata 18 Statistical Package (Stata Corp LLC, College-Station, TX, USA—2023). To explore the relationships between the response variables and indicators, we used multinomial logistic regression to report the relative risk ratio (RRR), logistic regression to report the odds ratio (OR), and linear regression. To test the independence, we used contingency tables with Fisher’s exact test. The significance of the models and predictors was assessed using validation parameters, with a *p*-value threshold of <0.05.

The mRNA expression of FBN1, FLNA, MMP2, and SOX9 were correlated with the histopathological features of the surgically removed valves within clinical parameters. The possible association between mRNA expressions and inflammatory parameters such as: LMR, CLR, PLR, PNR, and number of lymphocytes and monocytes, was also checked.

The study aims to explore potential causal relationships between the observed variables. In the statistical analysis, we primarily observed a significance level of *p* < 0.05. However, in some cases, we considered and mentioned results with a significance level of *p* < 0.1 to highlight trends that may be of potential interest for further investigation. The trends observed at the 0.1 level still offer valuable insights and potential directions for future research. While it is standard to use a significance level of 0.05 in many scientific analyses, there are contexts where a more lenient threshold, such as 0.1, is appropriate, especially in exploratory research or when dealing with smaller sample sizes. This choice is often justified in the literature to balance Type I and Type II errors. In Table 5, and in a few other instances, the significance level of 0.1 was explicitly mentioned and used to highlight trends that may not be strong enough to meet the 0.05 threshold but still indicate a noteworthy direction or effect. This was done to provide a more comprehensive understanding of the data and to identify potential areas for further investigation.

## 5. Conclusions

The critical role that genetic factors play in the development of mitral valve disorders, such as mitral valve prolapse and regurgitation, is profound and demands careful consideration. Mutations in a variety of genes that are responsible for encoding structural proteins, signaling molecules, and transcription factors—namely FBN1, FLNA, MMP2, and SOX9—have been identified as significant contributors to the pathology of mitral valve diseases. These genetic variations can disrupt the balance necessary for maintaining proper valve structure and function, leading to the deterioration of valve integrity and performance. FLNA and SOX9 seem to contribute to the aging of the valve and influence the hyalinization process, whereas inflammation and fibrosis are mediated by FBN1 and MMP2.

Incorporating genetic insights alongside clinical evaluations and advanced imaging techniques can significantly enhance our understanding of the mechanisms driving disease progression. This integrated approach not only helps in mapping out the complex interactions at the genetic level but also in identifying potential therapeutic targets for more effective treatment strategies.

Moreover, fostering interdisciplinary collaboration is essential for advancing our knowledge in this field. It is through the concerted efforts of clinicians, geneticists, and researchers that we can achieve a more comprehensive understanding of mitral valve pathology. Such collaborations are vital in developing innovative diagnostic tools and treatment modalities that can lead to improved patient outcomes. Emphasizing a multidisciplinary approach will help bridge gaps between research and clinical practice, ultimately benefiting those patients suffering from these challenging cardiac conditions.

## Figures and Tables

**Table 1:** Association between sex of the patient and mRNA expression. Fibrillin 1 (FNBN1), Filamin A (FLNA), matrix metalloproteinase 2 (MMP2), SRY-box transcription factor 9 (SOX9).

Gene	Females (*n* = 23)	Males (*n* = 31)	*p* Value
***FBN1***			0.007
low	4 (17.4%)	8 (34.8%)	
medium	8 (34.8%)	13 (41.9%)	
high	11 (47.8%)	10 (32.3%)	
***FLNA***			<0.0001
low	5 (21.7%)	9 (29%)	
medium	7 (30.4%)	16 (51.6%)	
high	11 (47.8%)	6 (9.14%)	
***MMP2***			<0.0001
low	11 (47.8%)	26 (83.9%)	
medium	12 (52.2%)	4 (12.9%)	
high	-	1 (3.2%)	
***SOX9***			1
low	-	2 (6.5%)	
medium	8 (34.8%)	9 (29%)	
high	15 (65.2%)	20 (64.5%)	

**Table 2:** Sex of the patient and age effect test on mRNA expression (Multinomial Logistic Regression, RRR). We consider the average expression (“normoexpression”) as the base level (for comparison). Fibrilin 1 (FNBN1), Filamin A (FLNA), matrix metalloproteinase 2 (MMP2), SRY-box transcription factor 9 (SOX9).

Gene	Expression	Sex/Age	P Stat	RRR	95% Confidence Interval	
FBN1	Low	Age	0.406	0.972	0.911	1.038
Prob > chi2 = 0.715		M	0.813	1.198	0.267	5.375
	High	Age	0.684	0.987	0.931	1.04
		M	0.342	0.550	0.160	1.888
FLNA	Low	Age	0.029	0.915	0.845	0.991
Prob > chi2 = 0.014		M	0.496	0.593	0.131	2.665
	High	Age	0.030	0.915	0.844	0.991
		M	0.020	0.179	0.042	0.761
MMP2	Low	Age	0.140	0.946	0.879	1.018
Prob > chi2 = 0.0079		M	**0.005**	7.104	1.811	27.864
	High	Age	0.803	1.040	0.759	1.427
		M	0.992	567	-	
SOX9	Low	Age	0.396	1.108	0.873	1.407
Prob > chi2 = 0.4871		M	0.993	816	-	
	High	Age	0.955	0.998	0.947	1.052
		M	0.779	1.182	0.368	3.798

**Table 3:** Correlation between mRNA expression for fibrilin 1 (FNBN1), filamin A (FLNA), matrix metalloproteinase 2 (MMP2), and SRY-box transcription factor 9 (SOX9) in blood sample and left ventricle ejection fraction (LVEF).

LVEF	Coefficient	Std. Err.	t	*p* > t	[95% Conf. Interval]	
FBN1						
Low	−0.388	2.556	−0.15	0.88	−5.537	4.760
High	−2.753	2.681	−1.03	0.31	−8.155	2.647
FLNA						
Low	−1.153	2.344	−0.49	0.625	−5.875	3.568
High	1.518	2.834	0.54	0.595	−4.190	7.228
MMP2						
Low	−1.687	2.024	−0.83	0.409	−5.765	2.390
High	**8.849**	3.394	2.61	**0.012**	2.012	15.685
SOX9						
Low	3.183	2.942	1.08	0.285	−2.743	9.110
High	−3.641	1.826	−1.99	0.052	−7.319	0.036

**Table 4:** Correlation between mRNA expression for fibrilin 1 (FNBN1), filamin A (FLNA), matrix metalloproteinase 2 (MMP2), and SRY-box transcription factor 9 (SOX9) from the valve tissue sample and left ventricle ejection fraction (LVEF). Assumed base level = normoexpression.

LVEF	Coefficient	Std. Err.	t	*p* > t	[95% Conf. Interval]	
FBN1						
Low	−9.292	2.942	−3.16	**0.003**	−15.219	−3.364
High	−3.706	2.897	−1.28	0.207	−9.542	2.129
FLNA						
Low	4.564	2.618	1.74	**0.088**	−0.709	9.839
High	1.555	3.164	0.49	0.625	−4.817	7.929
MMP2						
Low	5.708	2.169	2.63	**0.012**	1.338	10.078
High	3.0472	3.096	0.98	0.33	−3.188	9.283
SOX9valva						
Low	3.852	3.600	1.07	0.29	−3.399	11.104
High	−0.715	2.648	−0.27	0.788	−6.050	4.619

**Table 5:** The relationship between mRNA expression for fibrilin 1 (FNBN1), filamin A (FLNA), matrix metalloproteinase 2 (MMP2), and SRY-box transcription factor 9 (SOX9) from blood sample and inflammatory parameters—lymphocyte-monocyte ratio (LMR), C reactive protein-lymphocyte ratio (CLR), platelet-lymphocyte ratio (PLR), platelet-neutrophil ratio (PNR), lymphocytes and monocytes—analyzed using OLS regression. * Assumed base level = normoexpression.

LMR	Coef	SE	*p* > t	[95% Conf. Interval]		Observations
FBN1						
low	0.186	0.472	0.695	−0.766	1.138	
high	−1.017	0.454	**0.031**	−1.933	−0.100	The decrease in the LMR value is associated with the increased expression of FBN1 *
FLNA						
low	−0.396	0.459	0.393	−1.321	0.530	
high	0.242	0.418	0.565	−0.601	1.085	
MMP2						
low	0.080	0.365	0.828	−0.657	0.817	
high	0.984	1.192	0.414	−1.421	3.389	
SOX9						
low	−1.020	0.875	0.251	−2.786	0.747	
high	−0.066	0.346	0.848	−0.764	0.631	
**CLR**	**Coef**	**SE**	***p* > t**	**[95% Conf. Interval]**		
FBN1						
low	−0.505	0.458	0.279	−1.443	0.432	
high	−0.692	0.431	0.119	−1.575	0.190	
FLNA						
low	−0.662	0.443	0.146	−1.570	0.245	
high	−0.315	0.384	0.418	−1.101	0.470	
MMP2						
low	0.140	0.382	0.717	−0.642	0.922	
high	−0.004	0.994	0.997	−2.040	2.033	
SOX9						
low	0.072	0.721	0.921	−1.404	1.547	
high	−0.360	0.352	0.316	−1.081	0.361	
**PLR**	**Coef**	**SE**	***p* > t**	**[95% Conf. Interval]**		
FBN1						
low	11.366	22.456	0.615	−33.920	56.651	
high	6.796	21.598	0.755	−36.761	50.352	
FLNA						
low	19.267	21.835	0.382	−24.766	63.301	
high	10.705	19.682	0.589	−28.986	50.397	
MMP2						
low	−18.589	17.380	0.291	−53.638	16.460	
high	−38.284	56.734	0.503	−152.698	76.130	
SOX9						
low	−3.712	41.667	0.929	−87.741	80.317	
high	−2.054	16.439	0.901	−35.206	31.098	
**PNR**	**Coef**	**SE**	***p* > t**	**[95% Conf. Interval]**		
FBN1						
low	5.116	8.687	0.559	−12.414	22.647	
high	−10.332	8.124	0.210	−26.727	6.062	
FLNA						
low	−1.179	8.335	0.888	−17.999	15.642	
high	−2.104	7.498	0.780	−17.236	13.027	
MMP2						
low	3.034	6.376	0.637	−9.834	15.901	
high	5.623	21.448	0.794	−37.661	48.907	
SOX9						
low	−12.563	15.945	0.435	−44.741	19.615	
high	5.029	6.313	0.430	−7.711	17.769	
**Lymphocytes**	**Coef**	**SE**	***p* > t**	**[95% Conf. Interval]**		
FBN1						
low	0.206	0.293	0.485	−0.385	0.798	
high	−0.144	0.282	0.612	−0.713	0.424	
FLNA						
low	−0.147	0.285	0.608	−0.722	0.428	
high	−0.106	0.257	0.683	−0.624	0.412	
MMP2						
low	0.203	0.227	0.377	−0.255	0.660	
high	1.386	0.741	**0.068**	−0.109	2.880	The increase in the number of lymphocytes is associated with high levels of MMP2 * expression (significance at *p* < 0.1 level)
SOX9						
low	−0.466	0.544	0.397	−1.563	0.632	
high	0.065	0.215	0.764	−0.368	0.497	
**Monocytes**	**Coef**	**SE**	***p* > t**	**[95% Conf. Interval]**		
FBN1						
low	0.019	0.088	0.834	−0.159	0.197	
high	0.180	0.085	**0.040**	0.008	0.351	The increase in the number of monocytes is associated with high values of FBN1 *
FLNA						
low	0.031	0.086	0.721	−0.142	0.204	
high	−0.078	0.078	0.324	−0.236	0.079	
MMP2						
low	0.069	0.068	0.317	−0.068	0.207	
high	0.208	0.223	0.357	−0.242	0.6589	
SOX9						
low	0.017	0.164	0.919	−0.314	0.347	
high	−0.003	0.065	0.969	−0.133	0.128	

**Table 6:** Exploration using logistic regression, reporting Odds Ratio with and without adjustment (OR, adjusted-OR or aOR). Severity is expressed on two levels: low and high. Genetic expression is expressed on three levels: low, medium/or normal value, and high. Fibrilin 1 (FNBN1), Filamin A (FLNA), matrix metalloproteinase 2 (MMP2), SRY-box transcription factor 9 (SOX9). Assumed base level = normoexpression.

Fibrosis	OR	SE	*p*	[95% Conf. Interval]		aOR	SE	*p* > |z|	[95% Conf. Interval]	
FBN1										
low	2.308	2.120	0.363	0.381	13.963	0.895	1.079	0.927	0.084	9.498
high	9.231	10.499	0.051	0.993	85.774	9.825	14.267	0.116	0.570	169.179
FLNA										
low	1.875	1.724	0.494	0.309	11.372	5.829	8.009	0.199	0.394	86.113
high	2.344	2.134	0.350	0.393	13.963	2.847	3.838	0.438	0.202	39.991
MMP2										
low	7.333	5.873	0.013	1.5261	35.238	10.462	11.256	0.029	1.269	86.186
high	1.000	-				1.000	-			
SOX9										
low	1.000	-				1.000	-			
high	1.723	1.292	0.468	0.396	7.495	0.436	0.454	0.425	0.056	3.358
AGE						1.072	0.052	0.147	0.975	1.178
_cons						0.008	0.029	0.161	0.000	6.773
**Hyalinization**	**OR**	***p* > |z|**	***p***	**[95% conf. Interval]**		**aOR**	**SE**	***p* > |z|**	**[95% Conf. Interval]**	
FBN1										
low	0.700	0.554	0.652	0.148	3.301	0.291	0.327	0.272	0.032	2.627
high	2.133	1.692	0.339	0.450	10.097	5.980	6.709	0.111	0.663	53.909
FLNA										
low	1.250	1.017	0.784	0.253	6.162	9.883	12.145	0.062	0.889	109.869
high	1.125	0.844	0.875	0.2587	4.892	2.160	2.411	0.490	0.242	19.247
MMP2										
low	0.926	0.644	0.912	0.236	3.622	1.601	1.359	0.579	0.303	8.455
high	1.000	-				1.000	-			
SOX9										
low	0.214	0.332	0.321	0.010	4.476	0.044	0.089	0.121	0.001	2.277
high	0.548	0.410	0.421	0.126	2.371	0.182	0.180	0.085	0.026	1.264
AGE						1.105	0.052	0.035	1.006	1.212
_cons						0.006	0.018	0.105	0.0000	2.9173
**Myxoid** **Degeneration**	**OR**	***p* > |z|**	***p***	**[95% Conf. Interval]**		**aOR**	**SE**	***p* > |z|**	**[95% Conf. Interval]**	
FBN1										
low	0.196	0.161	0.047	0.039	0.981	0.164	0.184	0.108	0.018	1.483
high	1.000	0.786	1.000	0.214	4.666	0.518	0.521	0.513	0.071	3.729
FLNA										
low	0.565	0.419	0.441	0.131	2.417	2.671	3.125	0.401	0.269	26.463
high	1.647	1.308	0.530	0.347	7.806	2.112	2.036	0.438	0.319	13.969
MMP2										
low	1.364	0.904	0.640	0.372	4.997	2.289	1.844	0.304	0.471	11.105
high	1.000	-				1.000	-			
SOX9										
low	1.000	-				1.000	-			
high	1.818	1.246	0.383	0.474	6.964	1.550	1.198	0.571	0.340	7.047
AGE						1.019	0.034	0.568	0.954	1.089
_cons						0.528	1.288	0.793	0.004	63.199

**Table 7:** Correlation between mRNA expression in blood and tissue samples using Fisher Exact test.

Tissue	Blood	Fisher’s Exact Score
FBN1 tissue	FBN1 blood	0.000
FLNA tissue	FLNA blood	0.000
MMP2 tissue	MMP blood	0.000
SOX9 tissue	SOX9 blood	0.000

**Table 8:** The Fisher’s Exact test showing the correlation between the expression of FBN1, FNLA, MMP2 and SOX9 from tissue and blood sampling in different combinations.

Var 1	Var 2	Fisher’s Exact Score	Obs
FBN1 tissue	FLNA tissue	0.000	These parameters are associated
FBN1 tissue	MMP2 tissue	0.393	
FBN1 tissue	SOX9 tissue	0.136	
FLNA tissue	MMP2 tissue	0.305	
FLNA tissue	SOX9 tissue	0.120	
MMP2 tissue	SOX9 tissue	0.312	
FBN1 blood	FLNA blood	0.000	These parameters are associated
FBN1 blood	MMP2 blood	0.088	
FBN1 blood	SOX9 blood	0.017	These parameters are associated
FLNA blood	MMP2 blood	0.849	
FLNA blood	SOX9 blood	0.101	
MMP2 blood	SOX9 blood	0.602	

## Data Availability

The data included in the paper are available at the first author and can be obtained upon request.

## References

[1] Nkomo V.T., Gardin J.M., Skelton T.N., Gottdiener J.S., Scott C.G., Enriquez-Sarano M. (2006). Burden of valvular heart diseases: A population-based study. Lancet.

[2] Basso C., Iliceto S., Thiene G., Perazzolo Marra M. (2019). Mitral Valve Prolapse, Ventricular Arrhythmias, and Sudden Death. Circulation.

[3] Opris C.E., Suciu H., Flamand S., Opris C.I., Al Hussein H., Gurzu S. (2024). Update on the genetic profile of mitral valve development and prolapse. Pathol. Res. Pract..

[4] Gurzu S., Jung I. (2021). Subcellular expression of maspin in colorectal cancer: Friend or foe. Cancers.

[5] Elgammal R.M., Elsaiedy M.A., Alamrosy M.Z., Elsetiha M.E., Almasry M.M. (2023). Left ventricular assessment in patients with significant mitral incompetence: A multi-modality imaging study. J. Cardiol. Cardiovasc. Med..

[6] Aluru J.S., Barsouk A., Saginala K., Rawla P., Barsouk A. (2022). Valvular Heart Disease Epidemiology. Med. Sci..

[7] Patel A., Bapa V. (2017). Transcatheter mitral valve replacement: Device landscape and early results. EuroIntervention.

[8] Iung B., Baron G., Butchart E.G., Delahaye F., Gohlke-Bärwolf C., Levang O.W., Tornos P., Vanoverschelde J.-L., Vermeer F., Boersma E. (2003). A prospective survey of patients with valvular heart disease in Europe: The Euro Heart Survey on Valvular Heart Disease. Eur. Heart J..

[9] Otto C.M., Nishimura R.A., Bonow R.O., Carabello B.A., Erwin J.P., Gentile F., Jneid H., Krieger E.V., Mack M., McLeod C. (2021). 2020 ACC/AHA Guideline for the Management of Patients With Valvular Heart Disease: Executive Summary: A Report of the American College of Cardiology/American Heart Association Joint Committee on Clinical Practice Guidelines. Circulation.

[10] John S., Bashi V.V., Jairaj P.S., Muralidharan S., Ravikumar E., Rajarajeswari T., Krishnaswami S., Sukumar I.P., Rao P.S. (1983). Closed mitral valvotomy: Early results and long-term follow-up of 3724 consecutive patients. Circulation.

[11] Sullivan P.F., Fan C., Perou C.M. (2006). Evaluating the comparability of gene expression in blood and brain. Am. J. Med. Genet. B Neuropsychiatr. Genet..

[12] Battle A., Mostafavi S., Zhu X., Potash J.B., Weissman M.M., McCormick C., Haudenschild C.D., Beckman K.B., Shi J., Mei R. (2014). Characterizing the genetic basis of transcriptome diversity through RNA-sequencing of 922 individuals. Genome Res..

[13] Houseman E.A., Kim S., Kelsey K.T., Wiencke J.K. (2015). DNA Methylation in Whole Blood: Uses and Challenges. Curr. Environ. Health Rep..

[14] Bandaru S., Ala C., Zhou A.X., Akyürek L.M. (2021). Filamin A Regulates Cardiovascular Remodeling. Int. J. Mol. Sci..

[15] Peacock J.D., Levay A.K., Gillaspie D.B., Tao G., Lincoln J. (2010). Reduced sox9 function promotes heart valve calcification phenotypes in vivo. Circ. Res..

[16] Hinton R.B., Yutzey K.E. (2011). Heart valve structure and function in development and disease. Annu. Rev. Physiol..

[17] O’Donnell A., Yutzey K.E. (2020). Mechanisms of heart valve development and disease. Development.

[18] Kodigepalli K.M., Thatcher K., West T., Howsmon D.P., Schoen F.J., Sacks M.S., Breuer C.K., Lincoln J. (2020). Biology and Biomechanics of the Heart Valve Extracellular Matrix. J. Cardiovasc. Dev. Dis..

[19] Caja L., Dituri F., Mancarella S., Caballero-Diaz D., Moustakas A., Giannelli G., Fabregat I. (2018). TGF-β and the Tissue Microenvironment: Relevance in Fibrosis and Cancer. Int. J. Mol. Sci..

[20] Alonso F., Dong Y., Li L., Jahjah T., Dupuy J.W., Fremaux I., Reinhardt D.P., Génot E. (2023). Fibrillin-1 regulates endothelial sprouting during angiogenesis. Proc. Natl. Acad. Sci. USA.

[21] Peeters S., De Kinderen P., Meester J.A.N., Verstraeten A., Loeys B.L. (2022). The fibrillinopathies: New insights with focus on the paradigm of opposing phenotypes for both FBN1 and FBN2. Hum. Mutat..

[22] Mead T.J., Martin D.R., Wang L.W., Cain S.A., Gulec C., Cahill E., Mauch J., Reinhardt D., Lo C., Baldock C. (2022). Proteolysis of fibrillin-2 microfibrils is essential for normal skeletal development. eLife.

[23] Lin G., Tiedemann K., Vollbrandt T., Peters H., Batge B., Brinckmann J., Reinhardt D.P. (2002). Homo- and heterotypic fibrillin-1 and -2 interactions constitute the basis for the assembly of microfibrils. J. Biol. Chem..

[24] Yalim Z., Ersoy İ. (2022). Evaluation of inflammation markers in mitral valve prolapse. Arch. Cardiol. Mex..

[25] Visse R., Nagase H. (2003). Matrix metalloproteinases and tissue inhibitors of metalloproteinases: Structure, function, and biochemistry. Circ. Res..

[26] Irqsusi M., Mansouri A.L., Ramaswamy A., Rexin P., Salman M., Mahmood S., Mirow N., Ghazi T., Ramzan R., Rastan A.J. (2022). Role of matrix metalloproteinases in mitral valve regurgitation: Association between the of MMP-1, MMP-9, TIMP-1, and TIMP-2 expression, degree of mitral valve insufficiency, and pathologic etiology. J. Card. Surg..

[27] Kawagoe M., Tsuruga E., Oka K., Sawa Y., Ishikawa H. (2013). Matrix metalloproteinase-2 degrades fibrillin-1 and fibrillin-2 of oxytalan fibers in the human eye and periodontal ligaments in vitro. Acta. Histochem. Cytochem..

[28] Lemaître V., D’Armiento J. (2006). Matrix metalloproteinases in development and disease. Birth Defects Res. C Embryo Today.

[29] Kandhwal M., Behl T., Singh S., Sharma N., Arora S., Bhatia S., Al-Harrasi A., Sachdeva M., Bungau S. (2022). Role of matrix metalloproteinase in wound healing. Am. J. Transl. Res..

[30] Cancemi P., Aiello A., Accardi G., Caldarella R., Candore G., Caruso C., Ciaccio M., Cristaldi L., Di Gaudio F., Siino V. (2020). The Role of Matrix Metalloproteinases (MMP-2 and MMP-9) in Ageing and Longevity: Focus on Sicilian Long-Living Individuals (LLIs). Mediat. Inflamm..

[31] Janssens S., Lijnen H.R. (2006). What has been learned about the cardiovascular effects of matrix metalloproteinases from mouse models?. Cardiovasc. Res..

[32] Ronco D., Buttiglione G., Garatti A., Parolari A. (2023). Biology of mitral valve prolapse: From general mechanisms to advanced molecular patterns-a narrative review. Front. Cardiovasc. Med..

[33] Rabkin E., Aikawa M., Stone J.R., Fukumoto Y., Libby P., Schoen F.J. (2001). Activated interstitial myofibroblasts express catabolic enzymes and mediate matrix remodeling in myxomatous heart valves. Circulation.

[34] Akhtar S., Meek K.M., James V. (1999). Ultrastructure abnormalities in proteoglycans, collagen fibrils, and elastic fibers in normal and myxomatous mitral valve chordae tendineae. Cardiovasc. Pathol..

[35] Loardi C., Alamanni F., Trezzi M., Kassem S., Cavallotti L., Tremoli E., Pacini D., Parolari A. (2011). Biology of mitral valve prolapse: The harvest is big, but the workers are few. Int. J. Cardiol..

[36] Delling F.N., Vasan R.S. (2014). Epidemiology and pathophysiology of mitral valve prolapse: New insights into disease progression, genetics, and molecular basis. Circulation.

[37] Perrocheau M., Kiando S.R., Vernerey D., Dina C., Galan P., Hagege A., Jeunemaitre X., Bouatia-Naji N. (2015). Investigation of the Matrix Metalloproteinase-2 Gene in Patients with Non-Syndromic Mitral Valve Prolapse. J. Cardiovasc. Dev. Dis..

[38] Mahimkar R., Nguyen A., Mann M., Yeh C.C., Zhu B.Q., Karliner J.S., Lovett D.H. (2009). Cardiac transgenic matrix metalloproteinase-2 expression induces myxomatous valve degeneration: A potential model of mitral valve prolapse disease. Cardiovasc. Pathol..

[39] Mierziak J., Kostyn K., Boba A., Czemplik M., Kulma A., Wojtasik W. (2021). Influence of the Bioactive Diet Components on the Gene Expression Regulation. Nutrients.

[40] McDonagh T.A., Metra M., Adamo M., Gardner R.S., Baumbach A., Böhm M., Burri H., Butler J., Čelutkienė J., Chioncel O. (2023). 2023 Focused Update of the 2021 ESC Guidelines for the diagnosis and treatment of acute and chronic heart failure. Eur. Heart J..

[41] Kovacs Z., Banias L., Osvath E., Gurzu S. (2024). Synergistic impact of ARSB, TP53, and Maspin gene expressions on survival outcomes in colorectal cancer: A comprehensive clinicopathological analysis. Appl. Sci..

